# Dynamic Changes of Lipopolysaccharide Levels in Different Phases of Acute on Chronic Hepatitis B Liver Failure

**DOI:** 10.1371/journal.pone.0049460

**Published:** 2012-11-19

**Authors:** Calvin Pan, Yurong Gu, Wei Zhang, Yubao Zheng, Liang Peng, Hong Deng, Youming Chen, Lubiao Chen, Sui Chen, Min Zhang, Zhiliang Gao

**Affiliations:** 1 Division of Liver Diseases, Department of Medicine, The Mount Sinai Medical Center, Mount Sinai School of Medicine, New York, New York, United States of America; 2 Department of Infectious Disease, The Third Affiliated, Hospital of Sun-Yet-Sen University, Guangzhou, China; 3 Department of Infectious Disease, Beijing Youan Hospital, Capital Medical University, Beijing, China; Yonsei University College of Medicine, The Republic of Korea

## Abstract

**Background:**

High serum levels of lipopolysaccharide (LPS) with LPS-MD-2/TLR4 complex activated NF-kb and cytokine cause hepatic necrosis in animal models. We investigated the dynamic changes of LPS levels in patients with acute on chronic hepatitis B liver failure (ACHBLF).

**Methods:**

We enrolled ACHBLF patients for a 12-week study. Patients’ LPS levels were measured along with 10 healthy controls. Patients on supportive care and recovered without intervention(s) were analyzed. Patients’ LPS levels during the disease progression phase, peak phase, and remission phase were compared with healthy controls.

**Results:**

Among 30 patients enrolled, 25 who received interventions or expired during the study period were excluded from the analysis, five patients on supportive care who completed the study were analyzed. Significant abnormal distributions of LPS levels were observed in patients in different phases (0.0168±0.0101 in progression phase; 0.0960±0.0680 in peak phase; 0.0249±0.0365 in remission phase; and 0.0201±0.0146 in controls; respectively, p<0.05). The highest level of LPS was in the peak phase and significantly elevated when compared to controls (0.0201±0.0146 vs. 0.0960±0.0680, p = 0.007). There were no statistically significant differences in LPS levels between healthy controls and subjects in the progression phase or remission phase. Dynamic changes of LPS were correlated with MELD-Na in the progression phase (p = 0.01, R = 0.876) and in the peak phase (p = 0.000, R = −1.00).

**Conclusions:**

Significant abnormal distributions of LPS levels were observed in ACHBLF with the highest level in the peak phase. The dynamic changes of LPS were correlated with disease severity and suggested LPS causing secondary hepatic injury.

## Introduction

Hepatitis B virus (HBV) infection is the most common cause of liver disease worldwide [1]. Approximately 400 million people are suffering from chronic hepatitis B (CHB) infection and may develop complications like cirrhosis, and hepatocellular carcinoma (HCC) [2]. Acute on chronic liver failure (ACLF) is an acute hepatic insult in patients who have chronic liver disease, manifesting as jaundice (serum bilirubin>5 mg/dl or 85 mol/L) and coagulopathy (INR>1.5 or prothrombin activity<40%), often complicated by ascites and/or encephalopathy within 4 weeks of the acute presentation [3]. The underlying chronic liver diseases in ACLF vary depending on the geographic region. Alcoholic hepatitis is common in western countries, whereas chronic hepatitis B or C infections are often seen in Asian countries. The common participating factors include viral hepatitis reactivation, alcohol, hepatotoxic drugs/herbs. In acute on chronic hepatitis B liver failure (ACHBLF), HBV reactivation is the major acute insults and precipitation liver failure [3]. It may occur after withdrawal of HBV antiviral treatment but more often, due to non- HBV treatment related events, which include disease reactivation either spontaneous or secondary to intensive chemotherapy/immunosuppressive therapy. Liver transplantation is the only curative therapeutic option for ACHBLF with a 5-year survival rate of 85% [4], [5]. However, infectious complications often preclude transplant in patients with ACHBLF and many die on the waiting list due to the shortage of organs [6]. In 2008, the local standard of care for ACHBLF other than transplantation for ACHBLF was supportive care. Prior to the time we concluded this study, there was no prospective randomized control trial to support the effectiveness and safety use of antiviral therapy in patients with ACHBLF [7]. In addition, Lange et al reported that a significant portion of patients with high MELD scores and treated with entecavir developed lactic acidosis resulting in high mortality [8]. Thus, the local standard of care at that time required a detailed discussion with patients and obtaining the consent prior to the antiviral use in patients with ACHBLF. Due to the lacking of evidence on the use of antiviral for ACHBLF during our study period, two patterns of clinical practice were observed in our center: patients who believed the potential benefit of antiviral treatment were treated with nucleoside (tenofovir was not available in China), whereas, patients who believed that the antiviral had no role on hepatic regeneration during acute setting or unwilling to take the risk of lactic acidosis could defer the antiviral treatment until they recovered from the acute event, and then received antiviral treatment for CHB when their disease severity was improved (low MELD scores had less frequency of lactic acidosis). Our study was designed to capture those patients who deferred antiviral treatment but were able to recover spontaneously from ACHBLF without intervention.

The mechanism of ACHBLF remains unclear. It was speculated that pro-inflammatory cytokines mediated hepatic inflammation along with oxidative stress and the production of nitric oxide initiated the acute hepatic injury, followed by neutrophil dysfunction from circulating endotoxins (the cause of secondary liver damage), resulting in sepsis, multi-organ failure and impairment of liver regeneration [9], [10], [11], [12], [13]. LPS is an endotoxin derived from Gram-negative bacteria in the intestinal micro-flora. Evidently, trace amounts of LPS were measurable in serum samples from portal vein in normal healthy subjects since LPS may penetrate the intestinal mucosa. However, the majority of LPSs were cleared by liver filtration [10], [14]. West et al demonstrated that about 40%–50% of an intravenous dose of LPS was cleared up by the liver filtration in animal models [13]. In addition to the filtration, hepatic and Kupffer cell (KC) uptake in the liver with detoxification played a key role in preventing high circulating levels of LPS [9]. In CHB patients, Sozinov et al observed that high incidence of Gram-negative bacteria overgrowth leads to the over production of LPS and results in higher serum levels of LPS [14]. On the other hand, several studies in animal models suggested that delayed clearance of LPS from the circulation occurred in chronic liver diseases because of the impaired phagocytosis of KC [15], [16], [17]. The persistence of endotoxinemia not only activated the liver immune cells with participating inflammatory process but also caused dysfunction of liver parenchymal cells and apoptosis [18]. Another theory on hepatic injury implied that LPS in the circulation interacted with toll like receptor 4 (TLR4) and mediated a signal transduction pathway, which included the formation of LPS-LBP-CD14-secreted protein MD-2-TLR4 receptor complex [19], [20], [21]. The complex combined with myeloid differentiation factor 88, then phosphorylated and activated a series of cell kinases [21]. The activated kinases collectively further activated the transcription factor, mainly nuclear factor κB (NF-κB) [19], [22], which resulted in increased production of pro-inflammatory cytokines, and led to hepatic necrosis [19], [20], [21], [22], [23]. Lastly, LPS may also activate hepatic stellate cells (HSCs) to up-regulate gene expression of chemokines and adhesion molecules to induce liver injury [24], [25], [26].

Although the above theories on liver injury from LPS have been supported by animal models or a few in vivo studies, the relationship between the circulating LPS levels and liver disease activity or severity has not been fully explored in patients with ACHBLF. Previous published studies have focused on compensated liver disease or acute liver failure, which showed a significant correlation between elevated serum levels of LPS and liver disease severity [11], [14], [27]. In animal models for ACLF, Han et al suggested that LPS circulating in the blood may reach a certain level and then triggered the secondary liver injury on top of primary chronic liver disease. However, this theory has not been fully explored in patients with ACHBLF [10]. We sought to investigate LPS levels in different disease stages of ACHBLF and the dynamic changes of LPS levels associated with the disease severity measured by clinical parameters in ACHBLF patients.

## Study Design and Methods

This was a 12 week prospective, observational study with healthy controls that enrolled ACHBLF patients and healthy volunteers from a single tertiary care center, the Third Affiliated Hospital of Sun Yet-Sen University in China from October 2008 through April 2010. The study protocol and the inform consent form were both approved (IRB approval N0∶2008-321) by the Ethical Committee Board of Sun Yet-Sen University. All subjects were (or their designated health care proxy holders) consented prior to the screening.

### Study Population and Data Collection

Adult patients with ACHBLF who were willing to participate and consented to the study were screened for the following eligibility criteria: (1) age of 18–50 years; (2) meeting the diagnostic criteria of ACHBLF which included jaundice (serum bilirubin ≧5 mg/dl [85 umol/l]) and coagulopathy (INR ≧1.5 or prothrombin activity<40%), ascites and/or encephalopathy as determined by physical examination within 4 weeks of the disease onset, and previously diagnosed chronic hepatitis B. (3) exacerbation of CHB for the first time. Key exclusion criteria were the followings: the time point of acute onset of ACHBLF was more than 14 days prior to the enrollment date; clinical evidence of cirrhosis or documented stage IV fibrosis on liver biopsy (if available); co-infection with hepatitis A, C, D, E or HIV virus; pregnant woman; diagnosis of other liver diseases including autoimmune hepatitis and Wilson disease, or evidence of hepatic tumor; history of renal, cardiovascular, pulmonary, endocrine or neurological diseases; history of antiviral therapy prior to the onset of ACHBLF, history of drug abuse including alcohol abuse; treatment with immune modulator, antibiotic treatment, or Chinese herbal medicine within six months prior to the screening.

Patients enrolled were followed every week by research team until week 12. As per good clinical practice standard, further interventions for ACHBLF in addition to supportive care were allowed and decided by clinical team members who were blind to the protocol, which included referral for liver transplant, providing antiviral treatment or using antibiotic when sepsis developed. However, only patients who were on supportive care without interventions during the study period were analyzed to delineate the relationship between LPS levels and disease severity in ACHBLF. Total bilirubin (TBil) levels were used as the marker for disease phases in ACHBLF. According to the dynamic change of TBil, the phases of ACHBLF in this study were defined as the following: 1) progression phase, which was from the onset of ACHBLF (at the time of diagnosis of ACHBLF) to the point of peak level of TBil; 2) peak phase, which was the period when TBil level plateaued after reaching the peak; and 3) remission phase, which was from the point of decrease in TBil after plateauing to the return of TBil level to the baseline. Although clinical parameters were measured and LPS samples were obtained weekly, only 1–2 samples collected during each phase of ACHBLF (selected at the mid time point of the phase) were used to determine the LPS level in the individual phase.

Available serum and plasma samples were measured in our research laboratory. Patients’ HBV DNA levels, HBeAg and HBsAg status, ALT, albumin, creatinine, prothrombin time, model for end stage liver disease scores with sodium (MELD-Na) were recorded in all subjects at one week interval. Data for healthy volunteers were also prospectively collected and their blood samples were measured for LPS levels and TBil level in the same laboratory. The standard of supportive care for ACHBLF at the study center was the following: patients routinely received high calorie diet (35–40 Cal/kg/day) with reduced glutathione. Patients also received proton pump inhibitors, enteral/parenteral nutrition, and albumin transfusion if needed.

### Measurement of LPS and Parameters for Disease Severity

The primary measurement of this study was the followings: 1) measured serum LPS levels in three disease phases of ACHBLF and compared to those in the control group; 2) evaluated dynamic changes in LPS levels in ACHBLF by comparing the distribution pattern of LPS levels throughout all phases of ACHBLF. The secondary measurement was to assess the association between LPS levels and the disease severity indicated by MELD-Na scores. In addition to MELD-Na scores, serum total bilirubin levels were also selected as the main markers for the disease severity in this study. Other clinical parameters were also assessed, which included aspartate aminotransferase (AST), and alanine aminotransferase (ALT) albumin, creatinine (automatic biochemical analyzer, Olympus, Japan), prothrombin time (automatic hemostasis/thrombosis analyzer, STA compact).

All laboratory tests for patients in our center were performed in the hospital central laboratory. HBV serologies were tested by either CMIA (Abbott I 2000, USA) or ECL kits (Roche Laboratories, Germany). All serum samples for HBV DNA were tested by real-time quantitative PCR (Shanghai Kehua Bio-engineering Co., Ltd., China) with the detection range of 500 copies –8 log10 copies/mL. Total bilirubin (TBIL), ALT (ULN = 40 U/L) and other biochemistry markers were tested by a Hitachi 7600 fully-automatic biochemical (Hitachi Co. Ltd., Tokyo, Japan). LPS concentrations in plasma were measured with the Limulus Amebocyte Lysate (LAL) test (Xiamen Houshiji, Ltd., Xiamen, China) according to the manufacturer’s instructions. All the materials used were pyrogen free and LPS in the test samples was calculated by comparison to a standard curve. All samples were tested in duplicate and read at 450 nm for LPS in a Thermomax microplate reader (Molecular Devices, Sunnyvale, CA).

### Statistics

Baseline characteristics and laboratory results were summarized for two groups by means of descriptive statistics. SPSS 15.0 software (SPSS, Inc., Chicago, IL) was used to analyze changes within groups during the study period. Statistical significance between two groups was calculated using a one-way Anova test or nonparametric Mann-Whitney test. Correlation used the Pearson correlation test. The evaluation on the normality and pattern of LPS level distribution among three disease phases of ACHBLF were performed with Kolmogorov-Smirnov test and compared to LPS levels in the healthy controls. For all analyses, a p-value equal to or less than 0.05 was considered significant.

## Results

### 1. Clinical Characteristics and Baseline of Subjects

**Figure 1:**
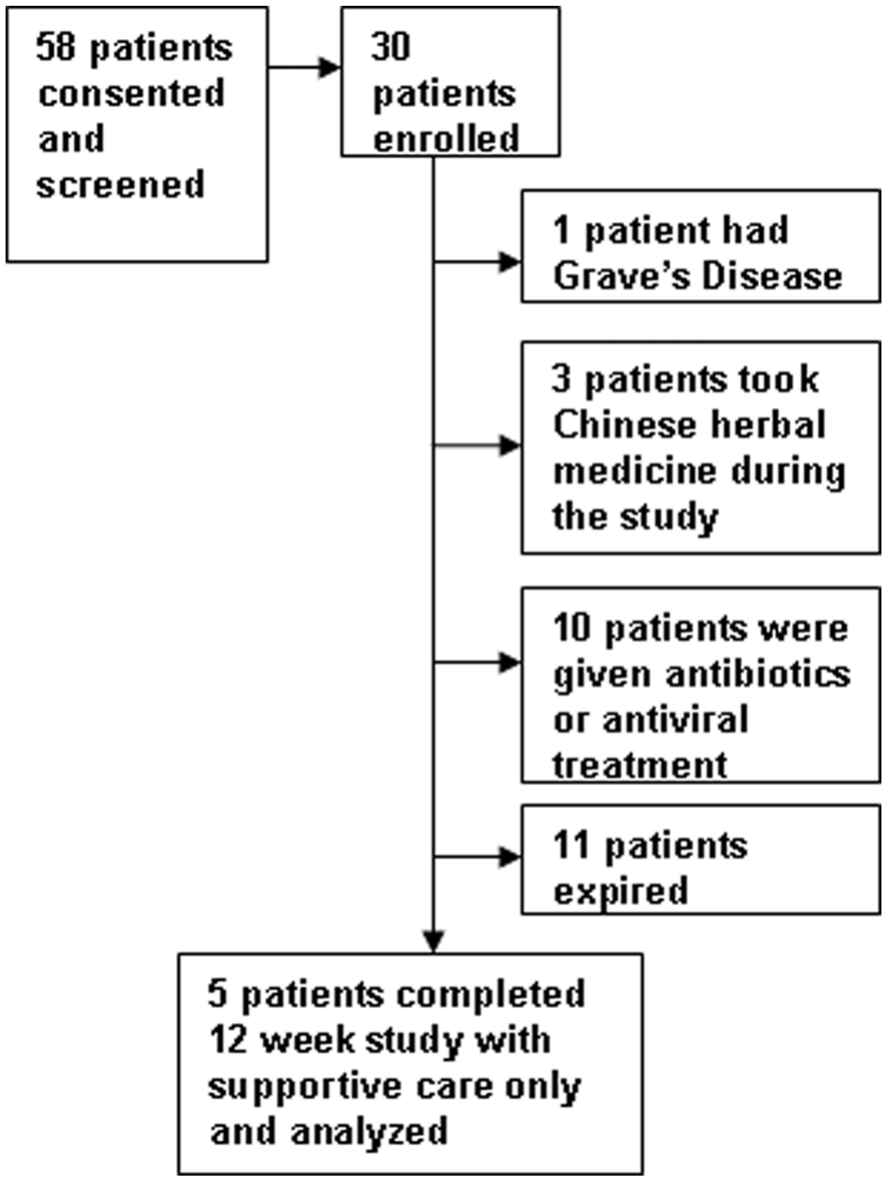
Patient selection and deposition.

Among 58 consecutive ACHBLF patients who consented and were screened with the above criteria, 30 patients enrolled. 25 patients were excluded from final analysis for the following reasons: 11 patients with rapid disease progression and died in the first 4 weeks (mostly from sepsis) despite interventions; 10 patients were excluded because of using antibiotics for infection or receiving antiviral therapy. 1 patient with CHB and history of Grave’s disease (history obtained after the enrollment) was suspected to have a flare of autoimmune hepatitis and received additional intervention; and 3 patients took herbs medication during the study period. A total of 5 patients who deferred antiviral treatment were included for the analysis and assigned to the ACHBLF group. These 5 patients had totally recovered from ACHBLF and were discharged after 12 to 16 weeks of hospitalization. A summary of patients’ deposition is shown in Figure 1. Ten healthy volunteers between the ages of 18 and 50 were enrolled and assigned to the control group. The baseline assessments of study subjects from these two groups are shown in Table 1. All five patients with ACHBLF were male and 80% were HBeAg positive. Their mean HBV DNA levels were 6.27±2.24 log10 IU/mL and mean MELD-Na scores were 19.22±3.8 at the baseline. All were admitted and observed with supportive care in the hospital and achieved remission without further intervention within 12 weeks. In addition, two female and eight male healthy volunteers were prospectively enrolled as controls. The mean ages of subjects in the two study groups were well matched without statistical significance.

**Table 1:** Baseline assessments of ACHBLF patients and healthy subjects.

Mean ± SD	Control group(n = 10)	ACHBLF group(n = 5)	case 1	case 2	case 3	case 4	case 5
**Male (M)**	8	5	M	M	M	M	M
**Age (year)** *	32.30±4.30	34.2±8.23	28	37	25	35	46
**HBeAg (%)**		(80%)	+	+	+	-	+
**HBV-DNA (log10 IU/mL)** *		6.27±2.24	3.44	6.22	8.39	4.71	8.56
**Serum bilirubin (umol/l)** *	12.33±2.06	307.54±78.53	237.1	321.7	215.8	389.8	373.3
**ALT (IU/l)** *	20.70±5.33	867±1004.88	423	921	2579	337	75
**AST (IU/l)** *	19.40±3.37	829.4±885.32	293	1466	2071	144	173
**Creatinine(mmol/l)** *	47.69±3.63	76.44±18.46	57.8	76.0	70.1	71.1	107.2
**Prothrobin time (Sec.)** *	12.54±0.51	26.4±4.11	23.3	33.2	23.7	24.5	27.3
**MELD-Na score**		19.22±3.8	15.13	25.00	17.67	20.14	17.55
**Serum LPS (EU/mL)**	0.0201±0.0146	0.0183±0.012	0.0086	0.0350	0.0095	0.0271	0.0113

Test of normality is done by Kolmogorov-Smirnov Test.

* P>0.05.

### 2. LPS Levels in Different Phases of ACHLF

**Figure 2:**
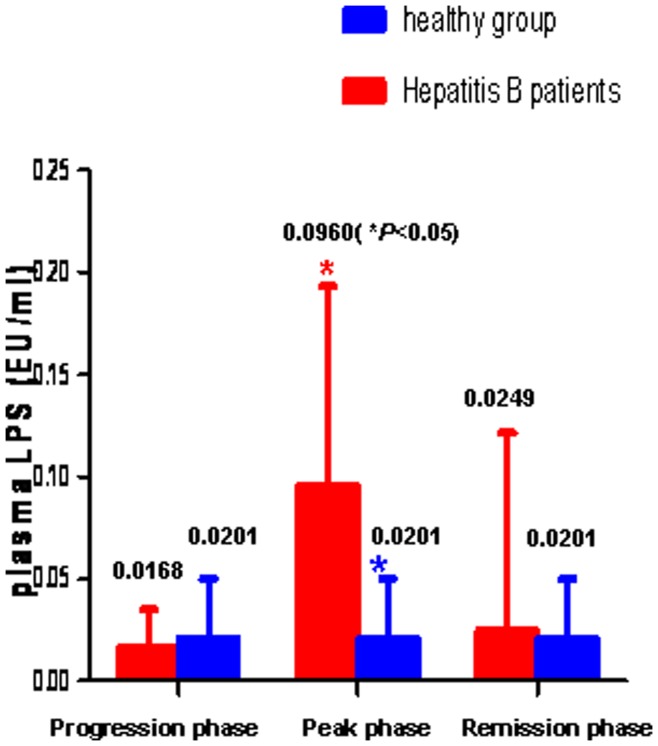
LPS levels in different disease phases compared to those in the healthy group.

In the ACHBLF group, the mean duration of disease progression from the onset of the disease to the peak phase was 31.24±2.77 days. The mean duration of the peak phase was 6.00±1.00 days. The mean duration of remission phase was 31.60±15.24 days. Significantly higher levels of LPS were observed in the peak phase compared to those in progression phase (0.0960±0.0680 vs. 0.0168±0.0101, p = 0.008) or those in remission phase (0.0960±0.0680 vs. 0.0249±0.0365, p = 0.021). When compared to the control group, LPS levels during the peak phase in ACHBLF were also significantly higher (0.0201±0.0146 vs. 0.0960±0.0680, P = 0.007). However, there were no statistically significant differences in LPS levels between controls and ACHBLF patients in progression phase (0.0168±0.0101 vs. 0.0201±0.0146, p = 0.618) or in remission phase (0.0249±0.0365 vs. 0.0201±0.0146, p = 0.706). The changes of LPS levels in different phases when compared to controls are shown in Figure 2.

### 3. The Dynamic Changes of LPS Levels and Disease Severity

The dynamic changes in mean levels of plasma LPS through the three phases of ACHBLF appeared to be a bell shape with a significant increase from the progressive phase to the peak phase, followed by a decrease or return to the baseline in the remission phase. Significantly abnormal distributions of LPS levels were observed during the phases of ACHBLF (Kolmogorov-Smirnov Test, P<0.05) when compared to healthy controls.

The highest MELD-Na mean scores in the ACHBLF group were observed in the peak phase and in parallel with the peak level of LPS. MELD-Na scores were correlated with LPS on progression phase (p = 0.01, R = 0.876) and peak phase (p = 0.000, R = −1.00). Although higher levels of TBil in patients with ACHBLF were observed in the peak phase and in parallel with the peak levels of LPS, the change of TBil were not statistically correlated with plasma LPS levels (p>0.05). The changes of LPS levels, Tbil and MELD-Na scores in the different phases of ACHBLF and their correlations are shown in Table 2.

**Table 2:** Total bilirubin, MELD-Na scores, and LPS levels in different phases of ACHBLF.

Mean ± SD	Progression phase	Peak phase	Remission phase
**Serum TBIL(umol/l)**	362.63±114.16	632.73±141.38	398.04±105.67
**MELD-Na score**	18.14±3.94*	26.35±18.23**	17.96±5.62
**Plasma LPS(EU/ml)**	0.0168±0.0101	0.096±0.068	0.0249±0.0365

MELD-Na score correlated with LPS in the progression phase (*p = 0.01, R = 0.876) and in the peak phase, (**p = 0.000, R = −1.00), respectively.

## Discussion

ACHBLF has significant morbidity and mortality with limited intervention unless liver transplant is offered [3], [5], [28]. However, even in the transplant center, majority of patients with ACHBLF die on the waiting list due to the shortage of organs or developed sepsis [3], [6]. The first prospective randomized control trial for antiviral use in ACHBLF patients was published by Grag et al in 2011, which demonstrated that tenofovir could safely reduce short term mortality in these patients. Our study was concluded prior to the first aforementioned randomized control trial. Although antiviral treatment may provide some short term survival benefits, many patients died despite the significant reduction of HBV DNA [29]. Thus, our data remained relevant for the understanding of disease mechanism and the future development of novel intervention. Previous studies have demonstrated that endotoxinemia and delayed clearance of LPS in the circulation resulted in the development of ACLF in alcoholic liver disease [18], [30], [31], [32], [33], [34]. Although Han et al proposed that LPS played an important role in ACHBLF as a secondary liver injury on top of the CHB infection in animal models [10]. The changes of LPS levels and their roles on disease severity in patients with ACHBLF were not fully explored.

Our study showed that baseline LPS levels in ACHBLF patients did not differ from those in the healthy controls. However, significant elevation in LPS levels was observed in the peak phase of ACHBLF when compared to those in the progression or remission phase.

The abnormal distributions of LPS levels among different phases were statistically significant in ACHBLF. In addition, the changes in LPS levels were correlated with MELD-Na scores in the progression and the peak phase. To our knowledge, this is by far the first study in which detailed the dynamic changes of LPS levels in different phases of ACHBLF, and provided the evidence of acute liver injury in ACHBLF associated with increased LPS levels. Since MELD-Na scores were correlated with LPS levels in the progression and the peak phase, our data pointed to the direction of the secondary injury from LPS in chronic liver disease leading to liver failure, which was proposed by Han et al. in the study from animal model. Further studies with histology correlation to LPS are needed to confirm if the severity of liver injury actually is directly correlated with LPS levels in ACHBLF patients. The findings in this study also implied a possible therapeutic intervention for ACHBLF by removing LPS from the serum.

Several studies done by Adachi et al observed that there was a positive correlation between the occurrence of bacterial translocation from the gut to portal system and liver dysfunction in alcoholic hepatitis [34], [35]. Li et al demonstrated that elevation of endotoxin levels in the circulation from translocation of gut flora occurred during acute flares in patients with chronic hepatitis [27]. It is possible that the elevation of LPS level in CHB patients was due to bacterial translocations from the gut to portal circulation resulting in endotoxemia in the early phase (or progressive phase ) of ACHBLF. On the other hand, the liver dysfunction in the early stage of ACHBLF probably further induced bacterial translocation from the gut leading to higher level of endotoxemia. In addition, in patients with liver dysfunction, the uptake of endotoxin by hepatic and Kupffer cells were compromised as compared to normal physical conditions, resulting in higher circulating levels of LPS [9], [13], [36]. High levels of LPS then induced the aggravations of liver injury through the LPS-MD-2/TLR4/NF-kb signal pathway and further negatively impacted on KC and hepatic clearance of endotoxin [33]. Thus, it is expected that the peak level of LPS was observed during the peak phase of ACHBLF. In our study, the dynamic changes of LPS were paralleled with the changes of TBil and MELD-Na in different phases of ACHBLF. The changes in LPS levels were correlated with MELD-Na scores in the progression and the peak phase, further indicated that the worsen disease severity was the result of LPS induced liver injury. The MELD-Na scores were not correlated with LPS levels in the remission phase. It is possible that the sample size of this study was too small to reflect such a correlation. Another possibility was that a delay on the improvement of MELD-Na scores occurred after the LPS level decreased by the buffering of LPS-binding substance produced in the remission phase. As suggested by previous studies, we presume that higher LPS levels were due to the production of LPS surpassed the phagocytic ability of kupffer cells rather than the decrease binding capacity of LPS-binding substances in acute phase [13], [36]. As disease progressed, the buffering system of LPS-binding substances was activated and reached the peak level in remission phase. Thus, it is possible that the liver injury induced by LPS was establish in the progression and peak phase with fluctuating lower levels of LPS-binding substances [12], [37]. Although our data was generated prospectively with control subjects, several limitations in this study are worth noting: 1) Patients included in the analysis were those who achieved spontaneous remission within 12 weeks on supportive care. The study result may not be applied to patients with prolonged peak phases and worsening disease activity or received additional intervention on top of supportive care; 2) Data analysis excluded patients expired during the study. Thus, the scale or levels of LPS in patients with severe liver necrosis remains uncertain. 3) For the feasibility of the study, we use healthy individuals as controls, which were less desirable than using CHB patients without ACHBLF. 4) A number of patients were not analyzed due to the intervention during the study period as required by the standard of care, such as antibiotic treatments for sepsis and antiviral use if patient consented to it. These patients were excluded because antibiotic may affect the gut flora and antiviral may influence the LPS level, which was reported by Koh et al in patients with CHB or hepatitis C during antiviral treatment [38]. Due to the limited numbers of patients that were analyzed in this study, a future trial with the larger sample size is warranted to confirm our findings.

In conclusion, the peak levels of LPS occurred during the severe necrosis phase (peak phase) in ACHBLF patients. The abnormal distributions of LPS levels among different phases were statistically significant in ACHBLF when compared to the controls. The highest MELD-Na mean scores in the ACHBLF group were observed in the peak phase and in parallel with the peak level of LPS. MELD-Na scores were correlated with LPS on progression phase and peak phase. Our data demonstrated the dynamic changes of LPS in ACHBLF as well as the relationship between LPS levels and the disease severity indicated by MELD-Na scores. These findings are important and may serve as the concept for the future development of therapeutic agents with the capacity to reduce LPS production or improve LPS clearance, which may have a significant impact on the clinical outcome of patients with ACHBLF.
